# Comparative Diagnostic Accuracy of AI-Assisted Fluorine-18 Fluorodeoxyglucose Positron Emission Tomography Versus Structural Magnetic Resonance Imaging in Alzheimer Disease: Systematic Review and Meta-Analysis

**DOI:** 10.2196/76981

**Published:** 2025-10-08

**Authors:** Bingbing Wang, Tailiang Zhao, Rongrong Ma, Xiaochuan Huo, Xiaoxiao Xiong, Minjie Wu, Yuran Wang, Liu Liu, Zhijiang Zhuang, Bin Wang, Jixin Shou

**Affiliations:** 1 Department of Neurosurgery, Fifth Affiliated Hospital of Zhengzhou University, No. 3 Kangfu-qian Street, Zhengzhou, 450052, China, 86 17630927442; 2 Department of Anesthesiology and Perioperative Medicine, Xuchang Central Hospital, Henan University of Science and Technology, Xuchang, China; 3 Department of Neurology, Fifth Affiliated Hospital of Zhengzhou University, Zhengzhou, China; 4 The Fifth Clinical Medical College, Henan Medical College, Zhengzhou University, Zhengzhou, China; 5 Department of Cardiology, Beijing Tsinghua Changgung Hospital, School of Clinical Medicine, Tsinghua University, Beijing, China

**Keywords:** ^18^F-FDG PET, Alzheimer disease, artificial intelligence, deep learning, machine learning, Preferred Reporting Items for Systematic Reviews and Meta-Analyses, PRISMA, sMRI, structural magnetic resonance imaging, systematic review

## Abstract

**Background:**

Neuroimaging is crucial in the diagnosis of Alzheimer disease (AD). In recent years, artificial intelligence (AI)–based neuroimaging technology has rapidly developed, providing new methods for accurate diagnosis of AD, but its performance differences still need to be systematically evaluated.

**Objective:**

This study aims to conduct a systematic review and meta-analysis comparing the diagnostic performance of AI-assisted fluorine-18 fluorodeoxyglucose positron emission tomography (^18^F-FDG PET) and structural magnetic resonance imaging (sMRI) for AD.

**Methods:**

Databases including Web of Science, PubMed, and Embase were searched from inception to January 2025 to identify original studies that developed or validated AI models for AD diagnosis using ^18^F-FDG PET or sMRI. Methodological quality was assessed using the TRIPOD-AI (Transparent Reporting of a Multivariable Prediction Model for Individual Prognosis or Diagnosis–Artificial Intelligence) checklist. A bivariate mixed-effects model was employed to calculate pooled sensitivity, specificity, and summary receiver operating characteristic curve area (SROC-AUC).

**Results:**

A total of 38 studies were included, with 28 moderate-to-high-quality studies analyzed. Pooled SROC-AUC values were 0.94 (95% CI 0.92‐0.96) for sMRI and 0.96 (95% CI 0.94‐0.98) for ^18^F-FDG PET, demonstrating statistically significant intermodal differences (*P*=.02). Subgroup analyses revealed that for machine learning, pooled SROC-AUCs were 0.89 (95% CI 0.86‐0.92) for sMRI and 0.95 (95% CI 0.92‐0.96) for ^18^F-FDG PET, while for deep learning, these values were 0.96 (95% CI 0.94‐0.97) and 0.97 (95% CI 0.96‐0.99), respectively. Meta-regression identified heterogeneity arising from study quality stratification, algorithm types, and validation strategies.

**Conclusions:**

Both AI-assisted ^18^F-FDG PET and sMRI exhibit high diagnostic accuracy in AD, with ^18^F-FDG PET demonstrating superior overall diagnostic performance compared to sMRI.

## Introduction

Alzheimer disease (AD) is a progressive neurodegenerative disorder characterized by insidious onset, cognitive decline, and memory impairment. In the United States, approximately 6.7 million adults aged ≥65 years are affected by AD, while China faces an even greater burden, with over 13 million cases [1,2]. The prolonged disease course and high comorbidity rates have established AD as one of the most fatal and economically burdensome conditions of the 21st century [3-5]. Accurate diagnosis of AD is critical for therapeutic decision-making and prognostic evaluation, particularly in the context of global population aging and increasing demands for precise patient stratification in clinical trials [6,7].

Neuroimaging modalities, including fluorine-18 fluorodeoxyglucose positron emission tomography (^18^F-FDG PET) and structural magnetic resonance imaging (sMRI), have become cornerstone technologies in AD diagnostic frameworks (eg, National Institute on Aging-Alzheimer's Association criteria) because of their noninvasive nature and quantitative capabilities [8-10]. Recent advancements in artificial intelligence (AI) have revolutionized medical image analysis: machine learning (ML) enables data-driven predictive modeling beyond traditional rule-based programming, while deep learning (DL), an advanced ML paradigm, employs multilayer neural networks to extract high-level features from complex datasets, demonstrating transformative potential in neuroimaging [11-14]. Despite extensive research on AI-assisted sMRI or ^18^F-FDG PET for AD diagnosis, two critical challenges hinder evaluation of model generalizability: (1) substantial heterogeneity in algorithm designs and validation frameworks, and (2) significant variability in the quality of individual studies [15]. Furthermore, no high-quality meta-analysis has comprehensively compared the diagnostic performance of these two imaging modalities.

This systematic review and meta-analysis addresses three critical objectives: (1) quantitative evaluation of diagnostic accuracy metrics for AI-assisted ^18^F-FDG PET and sMRI based on moderate-to-high-quality evidence; (2) direct comparison of diagnostic performance between modalities; and (3) investigation of confounding factors, including study quality (moderate-to-high vs low), algorithm types (ML vs DL), and validation strategies (internal vs external), through meta-regression. The findings are anticipated to inform evidence-based optimization of AD diagnostic pathways.

## Methods

### Overview

This study adhered to the PRISMA-DTA (Preferred Reporting Items for Systematic Reviews and Meta-Analyses of Diagnostic Test Accuracy) guidelines and was prospectively registered on PROSPERO (ID: CRD42023449927) to ensure transparency and minimize reporting bias [16,17]. Two reviewers independently conducted all stages of the review process, including title and abstract screening, full-text evaluation, data extraction, adherence assessment to reporting guidelines, and risk-of-bias evaluation. Discrepancies were resolved through group consensus.

### Literature Search Strategy

Two investigators (TLZ and BW) systematically searched PubMed, Web of Science, and Embase from inception to January 2025 using a combination of Medical Subject Headings (MeSH) terms and free-text keywords. Additional searches were performed on clinical trial registries and OpenGrey to identify unpublished clinical trials and gray literature. Search terms encompassed four domains: (1) disease terminology (Alzheimer disease, AD, Alzheimer Syndrome); (2) imaging modalities (^18^F-FDG PET, sMRI); (3) AI methodologies (ML, DL); and (4) diagnostic metrics (sensitivity, specificity, summary receiver operating characteristic curve area [SROC-AUC]). Reference lists of included studies were manually screened to identify additional relevant publications. The specific search strategies are provided in Table S1 in Multimedia Appendix 1.

### Literature Inclusion and Exclusion Criteria

The inclusion criteria were as follows: (1) human studies developing or validating AI models using ^18^F-FDG PET or sMRI to differentiate AD from normal controls; (2) AD diagnosis based on National Institute on Aging-Alzheimer's Association or International Working Group for New Research Criteria for Alzheimer’s Disease criteria; (3) availability of diagnostic performance metrics (eg, true positives, false positives, true negatives, false negatives) or explicit reporting of sensitivity and specificity; and (4) full-text availability in English. The exclusion criteria were as follows: (1) case reports, reviews, letters, or conference abstracts; (2) studies lacking sufficient diagnostic performance data; and (3) duplicate publications reporting on the same cohort without novel analyses.

### Literature Screening and Data Extraction

Two reviewers (TZ and BW) independently performed title and abstract and full-text screening, followed by cross-verification to ensure accuracy. Two additional investigators (RM and XH) extracted data using predefined forms, including study characteristics (first author, publication year), model specifications (algorithm type, validation strategies), and diagnostic performance metrics (2×2 contingency tables, sensitivity, specificity). Extracted data were cross-checked for completeness and precision.

### Quality Assessment of Included Studies

The methodological quality of included studies was evaluated using an adapted TRIPOD-AI (Transparent Reporting of a Multivariable Prediction Model for Individual Prognosis or Diagnosis–Artificial Intelligence) checklist (Table S2 in Multimedia Appendix 1) by two independent reviewers (RM and XH) [18]. The instrument assessed four domains: (1) data quality (sample diversity, adequacy, preprocessing standardization), (2) model development (feature extraction rationale, algorithm selection, hyperparameter optimization), (3) validation methods (cross-validation rigor, external validation), and (4) clinical applicability (interpretability, net clinical benefit). Each of the nine items was scored 0‐2 (0=unsatisfied; 1=partially satisfied; 2=fully satisfied), with total scores categorized as high quality (≥16), moderate quality (10-15), or low quality (≤9). Disagreements were resolved through discussion.

### Data Analysis

Diagnostic performance metrics were calculated using a bivariate mixed-effects model. SROC-AUC served as the primary outcome due to its threshold independence. Diagnostic accuracy was classified per National Institutes of Health criteria as high (AUC≥0.90), moderate (0.70‐0.89), or low (AUC<0.70) [19].

### Heterogeneity Assessment and Publication Bias Evaluation

Heterogeneity was quantified using Cochran *Q* test (significance defined at the .05 level) and *I*^2^ statistics (25%: low; 50%: moderate; 75%: high heterogeneity) [20]. Threshold effects were assessed using Spearman correlation between logit-transformed sensitivity and 1–specificity. Sensitivity analyses evaluated outlier influence by iteratively excluding individual studies. Univariable meta-regression analyses were conducted to assess the influence of potential confounding factors, including study quality (moderate to high vs low quality), algorithm type (ML vs DL), and validation strategy (internal vs external validation). Statistical significance of modifiers of the pooled effect size was defined at the .05 level after adjustment via the Holm-Bonferroni method. Publication bias was assessed via funnel plot asymmetry and Egger test, with significance defined at the .05 level. All analyses were conducted in Stata 16.0 using the MIDAS commands.

As this study focused on diagnostic accuracy, we employed the bivariate random-effects model via the MIDAS command in Stata to jointly synthesize sensitivity and specificity. This approach accounts for both within- and between-study variability, as well as the inherent correlation between sensitivity and specificity. In such models, conventional heterogeneity measures like *I*², although still reported for completeness, may not fully capture the joint variability of test performance measures. Therefore, to better understand heterogeneity, we additionally performed meta-regression and calculated joint likelihood ratio tests to explore potential effect modifiers. These methods align with recommended practices in diagnostic test accuracy meta-analyses [21,22].

## Results

### Literature Screening Process and Results

**Figure 1.**
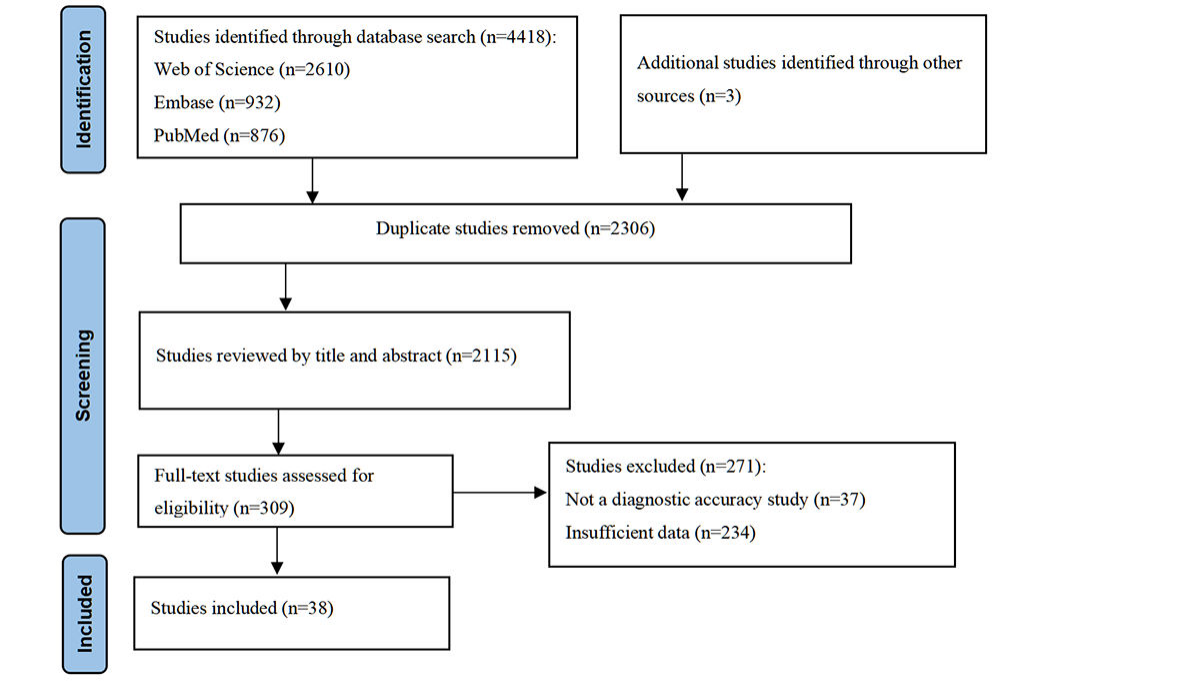
PRISMA (Preferred Reporting Items for Systematic Reviews and Meta-Analysis) flow diagram.

Initial searches identified 876 PubMed records (n=89 involving ^18^F-FDG PET; n=787 sMRI), 932 Embase records (n=111 ^18^F-FDG PET; n=821 sMRI), and 2610 Web of Science records (n=230 ^18^F-FDG PET; n=2380 sMRI), supplemented by 3 additional studies. After deduplication in EndNote 20, 2 reviewers independently screened titles and abstracts using the Population, Intervention, Comparison, Outcome, Study Design (PICOS) criteria, followed by full-text evaluation against predefined inclusion/exclusion criteria. This rigorous process yielded 38 studies for systematic review and meta-analysis [23-60]. The literature screening process and results are shown in Figure 1.

### Characteristics of Included Studies

Table 1 and Table S3 in Multimedia Appendix 1 detail the characteristics of the included studies. Imaging modalities comprised sMRI (n=15, 39%), ^18^F-FDG PET (n=15, 39%), and combined sMRI/^18^F-FDG PET (n=8, 22%). Data sources predominantly relied on open-access databases (n=29, 76%), with 28 (97%) from the Alzheimer’s Disease Neuroimaging Initiative database and 1 (3%) from the Open Access Series of Imaging Studies database. Four studies (11%) used institutional data, while 5 (13%) combined Alzheimer’s Disease Neuroimaging Initiative database with local datasets. Algorithmically, 26 studies (68%) employed ML, predominantly support vector machines (17/26, 65%), whereas 12 (32%) utilized DL, primarily convolutional neural networks (9/12, 75%). Internal validation was implemented in 33 studies (87%), with 10-fold cross-validation in 16 (48%); only 5 studies (13%) incorporated external validation.

**Table 1.** Characteristics of all included studies (n=38).

Studies	Model development and internal validation							Model external validation	
	Definition	Modality	Data source	Algorithm used	Training/validation (ratio)	Testing	Type of internal validation	Data source	Definition
Zhang et al [23], 2011	AD^a^: 51; NC^b^: 52	sMRI^c^; PET^d^	ADNI^e^ database	SVM^f^	NR^g^	NR	10-fold CV^h^	NR	NR
Yun et al [24], 2015	AD: 71; NC: 85	sMRI; PET	ADNI database	LDA^i^	NR	NR	LOOCV^j^	NR	NR
Westman et al [25], 2012	AD: 96; NC: 111	sMRI	ADNI database	OPLS^k^	NR	NR	7-fold CV	NR	NR
Vemuri et al [26], 2008	AD: 190; NC: 190	sMRI	Mayo Clinic	SVM	280	100	4-fold CV; hold-out	NR	NR
Suk et al [27], 2014	AD: 93; NC: 101	sMRI; PET	ADNI database	DBM^l^; SVM	NR	NR	10-fold CV	NR	NR
Sayeed et al [28], 2002	AD: 18; NC: 10	PET	Hammersmith Hospital	DFA^m^	NR	NR	LOOCV	NR	NR
Pan et al [29], 2019	AD: 247; NC: 246	PET	ADNI database	SVM	NR	NR	10-fold CV	NR	NR
Padilla et al [30], 2012	AD: 53; NC: 52	PET	ADNI database	SVM	NR	NR	LOOCV	NR	NR
Ni et al [31], 2021	AD: 638; NC: 629	PET	ADNI database	CNN^n^	1000	267	Hold-out	NR	NR
Magnin et al [32], 2009	AD: 16; NC: 22	sMRI	Pitié-Salpétriere Hospital	SVM	75%	25%	Bootstrap resampling	NR	NR
Lu et al [33], 2018	AD: 226; NC: 304	PET	ADNI database	MDNN^o^	NR	NR	10-fold CV	NR	NR
Liu et al [34], 2012	AD: 198; NC: 229	sMRI	ADNI database	SRC^p^	NR	NR	10-fold CV	NR	NR
Liu et al [35], 2018	AD: 93; NC: 100	PET	ADNI database	2D-CNN	NR	NR	10-fold CV	NR	NR
Li et al [36], 2015	AD: 44; NC: 45	PET	ADNI database; TUM database	GMM^q^; SVM	NR	NR	10-fold CV	NR	NR
Lerch et al [37], 2008	AD: 19; NC: 17	sMRI	Ludwig Maximilian University of Munich	QDA^r^	NR	NR	LOOCV	NR	NR
Kim et al [38], 2020	AD: 141; NC: 348	PET	ADNI database	CNN	NR	NR	NR	Severance dataset	AD: 80; NC: 72
Kim et al [39], 2020	AD: 139; NC: 347	PET	ADNI database	BEGAN^s^; SVM	NR	NR	NR	Severance dataset	AD: 73; NC: 68
Katako et al [40], 2018	AD: 94; NC: 111	PET	ADNI database	SVM	NR	NR	10-fold CV	NR	NR
Ismail et al [41], 2023	AD: 511; NC: 535	sMRI; PET	ADNI database	MultiAz-Net; SVM	NR	NR	10-fold CV	NR	NR
Illán et al [42], 2011	AD: 95; NC: 97	PET	ADNI database	SVM	NR	NR	LOOCV	NR	NR
Hinrichs et al [43], 2009	AD: 89; NC: 94	sMRI; PET	ADNI database	LPboosting^t^	NR	NR	Leave-many-out CV	NR	NR
Gray et al [44], 2012	AD: 50; NC: 54	PET	ADNI database	SVM	75%	25%	Monte Carlo CV	NR	NR
Gray et al [45], 2013	AD: 37; NC: 35	sMRI; PET	ADNI database	RF^u^	75%	25%	Monte Carlo CV	NR	NR
Feng et al [46], 2019	AD: 93; NC: 100	sMRI; PET	ADNI database	FSBi-LSTM^v^; 3D-CNN	NR	NR	10-fold CV	NR	NR
Cuingnet et al [47], 2011	AD: 137; NC: 162	sMRI	ADNI database	SVM	50%	50%	Hold-out	NR	NR
Chen et al [48], 2022	AD: 326; NC: 413	sMRI	ADNI-1 database; ADNI-2 database	2D-CNN; 3D-CNN	NR	NR	NR	ADNI-1 database; ADNI-2 database	AD: 326; NC: 413
Song et al [49], 2021	AD: 95; NC: 126	sMRI; PET	ADNI database	3D-CNN	NR	NR	10-fold CV	NR	NR
Li et al [50], 2019	AD: 130; NC: 162	PET	ADNI database	SVM	70%	30%	Monte Carlo CV	Huashan database	AD: 22; NC: 22
Ahila et al [51], 2022	AD: 220; NC: 635	PET	ADNI database	2D-CNN	90%	10%	Hold-out	NR	NR
Toussaint et al [52], 2012	AD: 80; NC: 80	PET	ADNI database	SVM	NR	NR	LOOCV	NR	NR
Tong et al [53], 2014	AD: 198; NC: 231	sMRI	ADNI database	SVM	NR	NR	LOOCV	NR	NR
Min et al [54], 2014	AD: 97; NC: 128	sMRI	ADNI database	SVM	NR	NR	10-fold CV	NR	NR
Jin et al [55], 2020	AD: 488; NC: 536	sMRI	In-house database; ADNI database	3D attention network	NR	NR	10-fold CV; leave center out CV	ADNI database; in-house database	AD: 488; NC: 536
Cho et al [56], 2012	AD: 128; NC: 160	sMRI	ADNI database	LDA	142	146	Hold-out	NR	NR
Chincarini et al [57], 2011	AD: 144; NC: 189	sMRI	ADNI database	RF; SVM	NR	NR	20-fold CV	NR	NR
Beheshti et al [58], 2017	AD: 92; NC: 94	sMRI	ADNI database	SVM	NR	NR	10-fold CV	NR	NR
Anandh et al [59], 2016	AD: 30; NC: 55	sMRI	OASIS^w^ database	SVM	NR	NR	10-fold CV	NR	NR
Amoroso et al [60], 2018	AD: 86; NC: 81	sMRI	ADNI database	RF	67	100	10-fold CV; hold-out	NR	NR

a AD: Alzheimer disease.

b NC: normal cognitive.

c sMRI: structural magnetic resonance imaging.

d PET: positron emission tomography.

e ADNI: Alzheimer’s Disease Neuroimaging Initiative.

f SVM: support vector machine.

g NR: no report.

h CV: cross-validation.

i LDA: linear discriminant analysis.

j LOOCV: leave-one-out cross-validation.

k OPLS: orthogonal partial least squares.

l DBM: deep Boltzmann machine.

m DFA: discriminant function analysis.

n CNN: convolutional neural network.

o MDNN: multiscale deep neural network.

p SRC: sparse representation–based classifier.

q GMM: Gaussian mixture model.

r QDA: quadratic discriminant analysis.

s BEGAN: boundary equilibrium generative adversarial network.

t LP: linear program.

u RF: random forest.

v FSBi-LSTM: fully stacked bidirectional long short-term memory.

w OASIS: Open Access Series of Imaging Studies.

### Quality Assessment Results of Included Studies

Nine (24%) studies were high quality, characterized by adequate sample sizes, rigorous validation (eg, multicenter external validation), and standardized reporting. Moderate-quality studies (n=19, 50%) met basic methodological standards (eg, cross-validation) but had limitations such as single-center data or insufficient clinical correlation analyses. Ten (26%) studies were low quality due to small sample sizes (<100 cases) and inadequate validation strategies, compromising external validity. The results of quality assessments for each study are detailed in Table S4 in Multimedia Appendix 1.

### Data Analysis Results

#### Overall Diagnostic Performance

Analysis of 29 sMRI-related contingency tables demonstrated a pooled sensitivity of 88% (95% CI 86%-89%), a specificity of 92% (95% CI 90%-93%), and an SROC-AUC of 0.94 (95% CI 0.92-0.96). Subgroup analyses stratified by algorithm type revealed that ML (19 tables) achieved a sensitivity of 86% (95% CI 85%-88%), a specificity of 91% (95% CI 88%-93%), and an SROC-AUC of 0.88 (95% CI 0.85-0.90), while DL (10 tables) showed improved performance with a sensitivity of 88% (95% CI 86%-91%), a specificity of 92% (95% CI 90%-94%), and an SROC-AUC of 0.96 (95% CI 0.94-0.97). Stratification by study quality indicated that moderate-to-high-quality studies (24 tables) yielded a sensitivity of 87% (95% CI 85%-89%), a specificity of 91% (95% CI 89%-93%), and an SROC-AUC of 0.94 (95% CI 0.92-0.96), whereas low-quality studies (5 tables) reported elevated metrics with a sensitivity of 91% (95% CI 87%-94%), a specificity of 95% (95% CI 92%-97%), and an SROC-AUC of 0.98 (95% CI 0.96-0.99). Validation strategy–based analysis showed that internal validation (25 tables) achieved a sensitivity of 88% (95% CI 86%-90%), a specificity of 92% (95% CI 90%-94%), and an SROC-AUC of 0.95 (95% CI 0.92-0.96), while external validation (4 tables) demonstrated marginally lower performance with a sensitivity of 85% (95% CI 81%-89%), a specificity of 91% (95% CI 85%-94%), and an SROC-AUC of 0.93 (95% CI 0.90-0.95).

For ^18^F-FDG PET (27 tables), pooled estimates demonstrated a sensitivity of 90% (95% CI 88%-92%), a specificity of 93% (95% CI 91%-94%), and an SROC-AUC of 0.96 (95% CI 0.94-0.98). Subgroup analyses stratified by algorithm type revealed that ML (15 tables) achieved a sensitivity of 89% (95% CI 86%-90%), a specificity of 91% (95% CI 88%-93%), and an SROC-AUC of 0.94 (95% CI 0.91-0.96), while DL (12 tables) exhibited superior performance with a sensitivity of 91% (95% CI 89%-93%), a specificity of 94% (95% CI 93%-96%), and an SROC-AUC of 0.98 (95% CI 0.96-0.99). Moderate-to-high-quality studies (21 tables) demonstrated a sensitivity of 90% (95% CI 88%-92%), a specificity of 93% (95% CI 91%-94%), and an SROC-AUC of 0.96 (95% CI 0.94-0.98), whereas low-quality studies (6 tables) showed comparable metrics with a sensitivity of 91% (95% CI 86%-94%), a specificity of 93% (95% CI 87%-96%), and an SROC-AUC of 0.96 (95% CI 0.94-0.98). Internal validation (24 tables) yielded a sensitivity of 90% (95% CI 89%-92%), a specificity of 93% (95% CI 91%-94%), and an SROC-AUC of 0.96 (95% CI 0.94%-0.97%), while external validation data (3 tables) were insufficient for SROC-AUC calculation but reported a sensitivity of 87% (95% CI 81%-93%) and a specificity of 95% (95% CI 91%-97%). The data analysis results are shown in Table 2.

**Table 2.** Summary estimates and meta-regression of pooled performance of all studies.

Main and subordinate directory		Tables, n	Sensitivity (%; 95% CI)	*I* ^2^	Specificity (%; 95% CI)	*I* ^2^	Joint *P* value	AUC^a^ (95% CI)
sMRI^b^								
Overall		29	88 (86-89)	55.32	92 (90-93)	74.08	—^j^	0.94 (0.92-0.96)
Study quality							.02	
Medium-to-high quality		24	87 (85-89)	57.99	91 (89-93)	75.94		0.94 (0.92-0.96)
Low quality		5	91 (87-94)	10.2	95 (92-97)	0		0.98 (0.96-0.99)
Algorithm type							.40	
ML^d^		19	86 (85-88)	21.23	91 (88-93)	73.82		0.88 (0.85-0.90)
DL^e^		10	88 (86-91)	76.56	92 (90-94)	76.53		0.96 (0.94-0.97)
Validation strategy							.33	
Internal validation		25	88 (86-90)	46.38	92 (90-94)	71.65		0.95 (0.92-0.96)
External validation		4	85 (81-89)	72.26	91 (85-94)	83.65		0.93 (0.90-0.95)
SVM^f^		10	87 (85-90)	17.47	93 (90-95)	57.65		0.93 (0.90-0.95)
CNN^g^		9	88 (85-91)	78.19	92 (90-94)	77.55		0.96 (0.93-0.97)
^18^F-FDG PET^k^								
Overall		27	90 (88-92)	42.04	93 (91-94)	61.49	—	0.96 (0.94-0.98)
Study quality							.96	
Medium-to-high quality		21	90 (88-92)	44.58	93 (91-94)	58.52		0.96 (0.94-0.98)
Low quality		6	91 (86-94)	42.73	93 (87-96)	72.3		0.96 (0.94-0.98)
Algorithm type							.01	
ML		15	89 (86-90)	1.61	91 (88-93)	45.64		0.94 (0.91-0.96)
DL		12	91 (89-93)	55.23	94 (93-96)	45.92		0.98 (0.96-0.99)
Validation strategy							.26	
Internal validation		24	90 (89-92)	34.61	93 (91-94)	64.9		0.96 (0.94-0.97)
External validation		3	87 (81-93)	—	95 (91-99)	—		—
SVM		11	90 (87-92)	0	92 (89-94)	37.5		0.94 (0.92-0.96)
CNN		8	91 (88-94)	69.76	93 (92-95)	34.63		0.97 (0.95-0.98)

a AUC: area under the receiver operating characteristic curve.

b sMRI: structural magnetic resonance imaging.

c Not applicable.

d ML: machine learning.

e DL: deep learning.

f SVM: support vector machine.

g CNN: convolutional neural network.

h 18F-FDG PET: fluorine-18 fluorodeoxyglucose positron emission tomograph.

#### Moderate-to-High-Quality Study Subgroup Analysis

**Figure 2.**
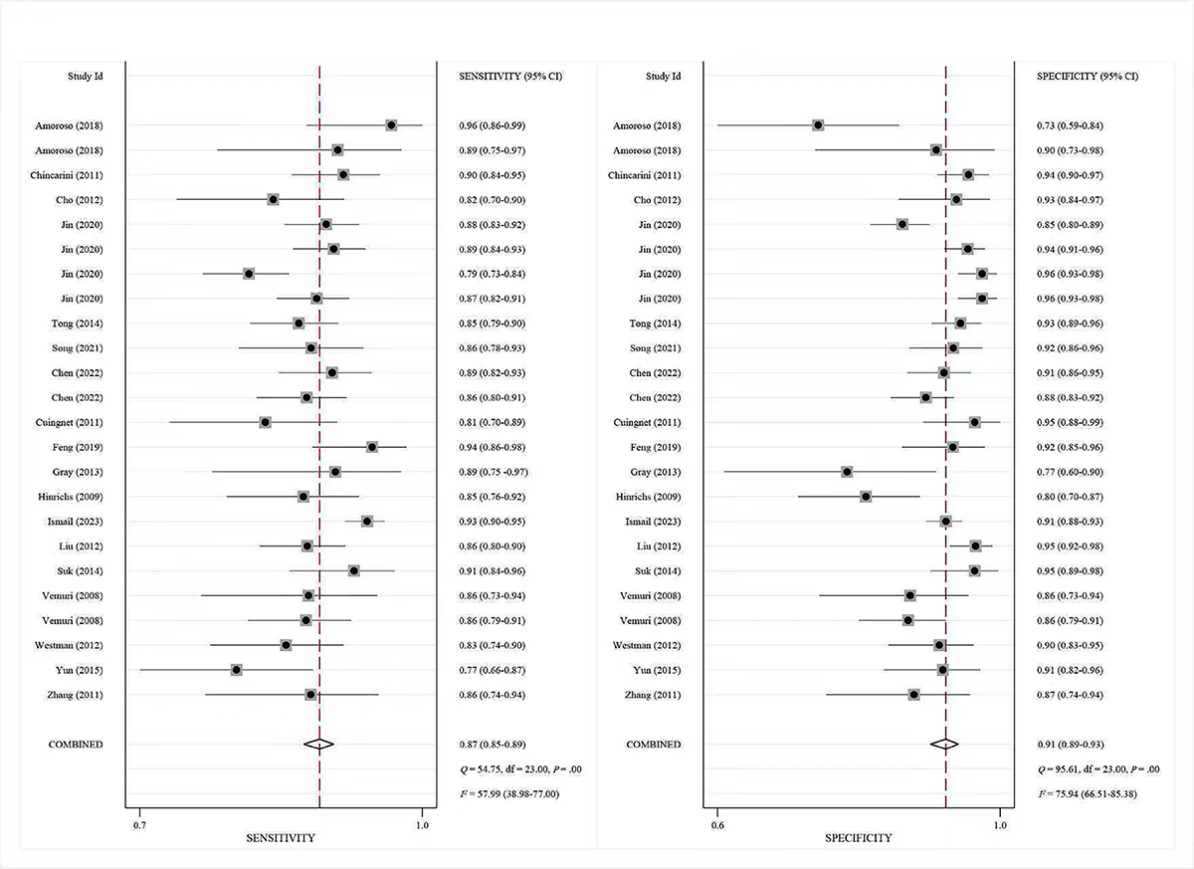
The pooled sensitivities and specificities based on moderate-to-high-quality studies for structural magnetic resonance imaging [23-27,34,41,43,45-49,53,55,57,60].

For sMRI, pooled sensitivity, specificity, and SROC-AUC were 87% (95% CI 85%‐89%), 91% (95% CI 89%‐93%), and 0.94 (95% CI 0.92‐0.96), respectively (see Figures2  3). Stratification by algorithm type indicated that ML models achieved 86% (95% CI 84%‐88%) sensitivity, 89% (95% CI 86%‐92%) specificity, and an SROC-AUC of 0.89 (95% CI 0.86‐0.92), while DL models demonstrated 88% (95% CI 86%‐91%) sensitivity, 92% (95% CI 90%‐94%) specificity, and an SROC-AUC of 0.96 (95% CI 0.94‐0.97).

For ^18^F-FDG PET, pooled sensitivity, specificity, and SROC-AUC were 90% (95% CI 88%‐92%), 93% (95% CI 91%‐94%), and 0.96 (95% CI 0.94‐0.98), respectively (see Figures4  5). Subgroup analyses revealed ML models achieved 89% (95% CI 86‐91%) sensitivity, 91% (95% CI 87%‐93%) specificity, and an SROC-AUC of 0.95 (95% CI 0.92‐0.96), whereas DL models outperformed with 91% (95% CI 89%‐93%) sensitivity, 94% (95% CI 93%‐96%) specificity, and SROC-AUC 0.97 (95% CI 0.96‐0.99). The data analysis results are also shown in Table 3.

**Figure 3.**
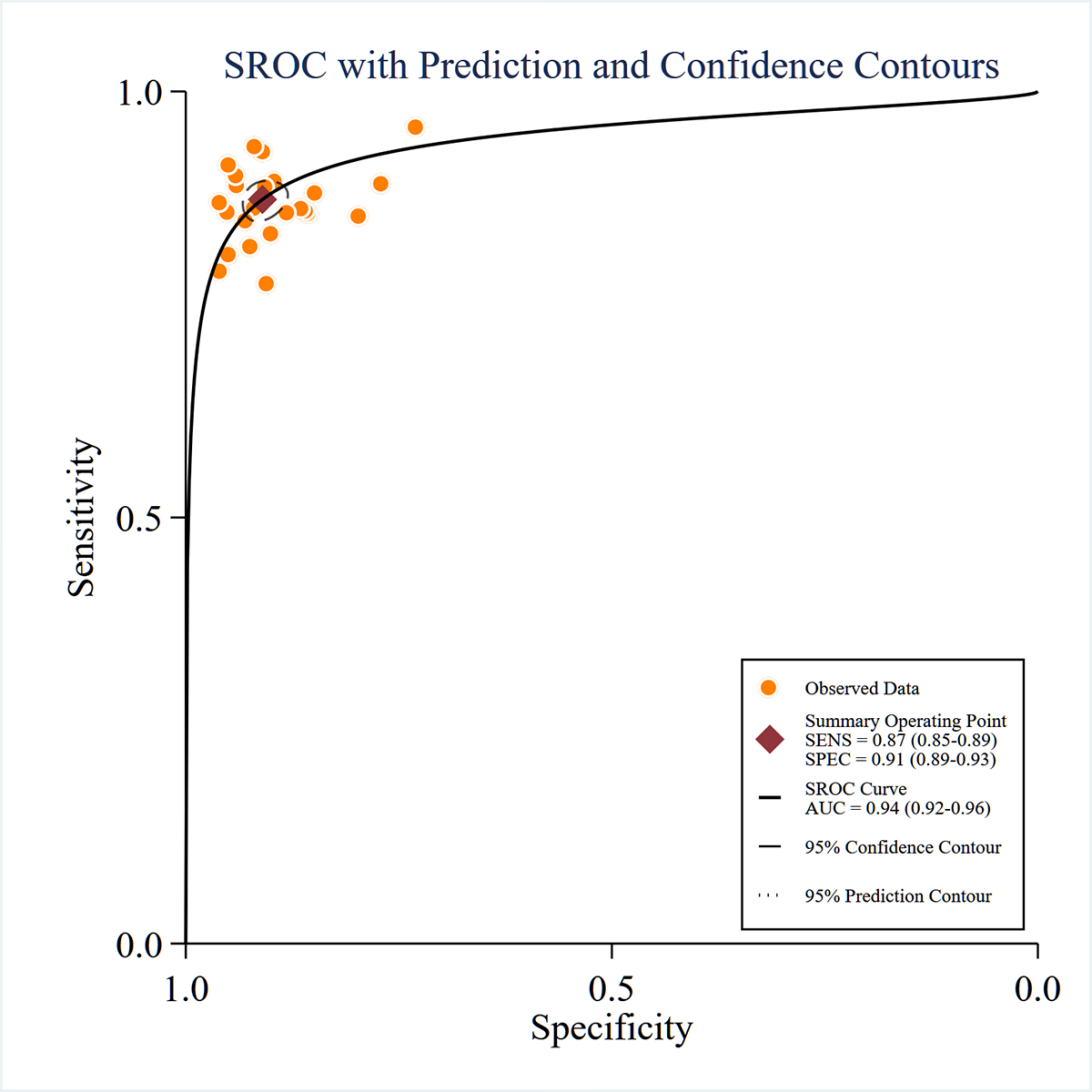
Summary receiver operating characteristic (SROC) based on moderate-to-high-quality studies curves for structural magnetic resonance imaging. AUC: area under the receiver operating characteristic curve area; SENS: sensitivity; SPEC: specificity.

**Figure 4.**
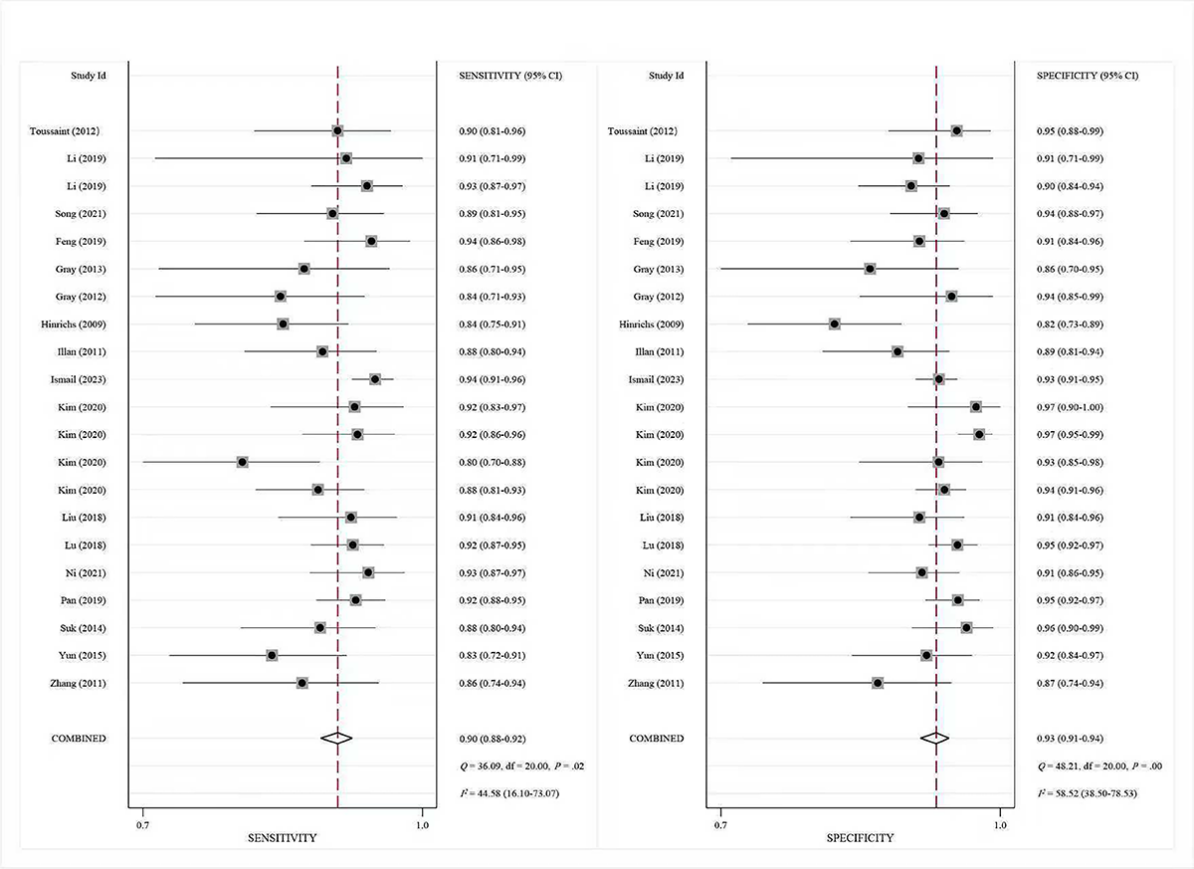
The pooled sensitivities and specificities based on moderate-to-high-quality studies for fluorine-18 fluorodeoxyglucose positron emission tomography (^18^F-FDG PET) [23,24,27,29,31,33,35,38,39,41-45,45,46,49,50].

**Figure 5.**
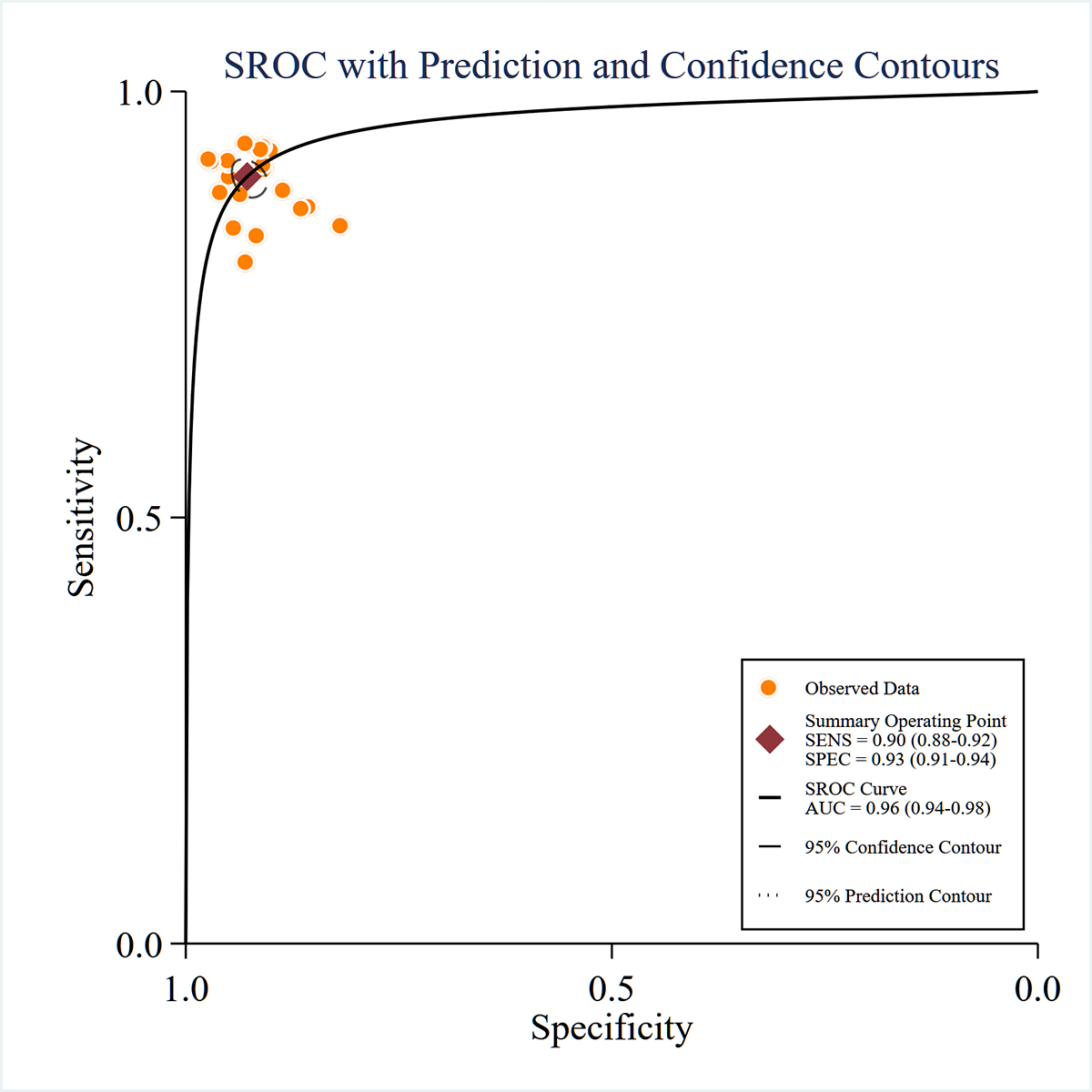
Summary receiver operating characteristic (SROC) based on moderate-to-high-quality studies curves for fluorine-18 fluorodeoxyglucose positron emission tomography (^18^F-FDG PET). AUC: area under the receiver operating characteristic curve area; SENS: sensitivity; SPEC: specificity.

**Table 3.** Summary estimates and meta-regression of pooled performance of moderate-to-high-quality studies.

Main and subordinate directory		Tables, n	Sensitivity (%; 95% CI)	*I* ^2^	Specificity (%; 95% CI)	*I* ^2^	Joint *P* value	AUC^a^ (95% CI)
Overall							.02	
sMRI^b^		24	87 (85-97)	57.99	91 (89-93)	75.94		0.94 (0.92-0.96)
^18^F-FDG PET^c^		21	90 (88-92)	44.58	93 (91-94)	58.52		0.96 (0.94-0.98)
ML^d^								
sMRI		14	86 (84-88)	3.72	89 (86-92)	75.58		0.89 (0.86-0.92)
^18^F-FDG PET		10	89 (86-91)	16.51	91 (87-93)	53.95		0.95 (0.92-0.96)
DL^e^								
sMRI		10	88 (86-91)	76.56	92 (90-94)	76.53		0.96 (0.94-0.97)
^18^F-FDG PET		11	91 (89-93)	54.59	94 (93-95)	36.23		0.97 (0.96-0.99)

a AUC: area under the receiver operating characteristic curve.

b sMRI: structural magnetic resonance imaging.

c 18F-FDG PET: fluorine-18 fluorodeoxyglucose positron emission tomography.

d ML: machine learning.

e DL: deep learning.

### Exploration of Statistical Heterogeneity Sources

#### Full Study Cohort

For sMRI contingency tables, sensitivity exhibited moderate heterogeneity (*I*²=55.32%), while specificity showed high heterogeneity (*I*²=74.08%). Threshold effect testing revealed no significant threshold effect (*r*=−0.101, *P*=.60). Subgroup analyses identified study quality stratification (*P*=.02) as a source of heterogeneity, with moderate-to-high-quality studies demonstrating lower sensitivity (87% vs 91%) and specificity (91% vs 95%) compared to low-quality studies. Algorithm type (*P*=.40) and validation strategy (*P*=.33) were not significant contributors.

For ^18^F-FDG PET analyses, sensitivity and specificity displayed moderate (*I*²=42.04%) and high heterogeneity (*I*²=61.49%), respectively, with no threshold effect (*r*=−0.087, *P*=.67). Algorithm type (*P*=.01) significantly influenced heterogeneity, as ML models demonstrated lower sensitivity (89% vs 91%) and specificity (91% vs 94%) than DL. Study quality (*P*=.96) and validation strategy (*P*=.26) showed no significant impact. The data analysis results are also shown in Table 2.

#### Moderate-to-High-Quality Study Subgroups

For sMRI-based models, ML implementations demonstrated minimal sensitivity heterogeneity (*I*²=3.72%) but high specificity heterogeneity (*I*²=75.58%). Exclusion of studies using traditional ensemble algorithms (random forest, boosting) reduced specificity heterogeneity to moderate (*I*²=45.47%), revealing that ensemble methods achieved higher sensitivity (89% vs 87%) but significantly lower specificity (79% vs 93%) compared to nonensemble ML models (*P*<.001). In contrast, DL-based sMRI models exhibited high heterogeneity for both sensitivity (*I*²=76.56%) and specificity (*I*²=76.53%). Removing external validation data mitigated heterogeneity to moderate (*I*²=54.36%) and low levels (*I*²=38.60%), respectively, with external validation studies showing significantly reduced sensitivity (85% vs 90%) and specificity (91% vs 93%) compared to internal validation (*P*<.001).

For ^18^F-FDG PET-based models, ML implementations showed low sensitivity heterogeneity (*I*²=16.51%) and moderate specificity heterogeneity (*I*²=53.95%). DL models exhibited moderate sensitivity heterogeneity (*I*²=54.59%) and low specificity heterogeneity (*I*²=36.23%). The data analysis results also are shown in Table 3 and Table S5 in Multimedia Appendix 1.

### Publication Bias Test

Funnel plot analysis of the full study cohort revealed no significant publication bias for sMRI (*P*=.69), whereas the ^18^F-FDG PET subgroup exhibited significant bias (*P*<.001). In the moderate-to-high-quality cohort, both sMRI (*P*=.03) and ^18^F-FDG PET (*P*=.01) demonstrated publication bias. However, subgroup analyses showed no significant bias for sMRI+ML (*P*=.06), sMRI+DL (*P*=.89), ^18^F-FDG PET+ML (*P*=.08), or ^18^F-FDG PET+DL (*P*=.28).

## Discussion

### Principal Findings

This meta-analysis confirms that AI-enhanced sMRI and ^18^F-FDG PET achieve high diagnostic accuracy for AD, with pooled SROC-AUCs of 0.94 and 0.96, respectively, outperforming conventional visual assessments [61,62]. ^18^F-FDG PET demonstrated superior overall performance (*P*=.02), likely attributable to its sensitivity to AD-specific metabolic abnormalities. Notably, ML amplified PET’s diagnostic advantage (SROC-AUC: 0.95 vs 0.89), while DL narrowed the gap (SROC-AUC: 0.97 vs 0.96), highlighting DL’s capacity to extract complex metabolic features and mitigate structural imaging limitations [63].

Subgroup analyses identified three key determinants of heterogeneity and performance variation. First, methodological quality significantly influenced sMRI models: moderate-to-high-quality studies reported lower sensitivity (87% vs 91%) and specificity (91% vs 95%) compared to low-quality studies (*P*=.02), suggesting inflated performance in the latter due to small sample sizes, single-center data, or nonstandardized preprocessing. Adherence to TRIPOD-AI guidelines and transparent reporting of preprocessing workflows are critical for future studies [18]. Second, algorithm type drove technical divergence in PET models: DL outperformed ML (sensitivity: 91% vs 89%; specificity: 94% vs 91%; *P*=.01) through end-to-end feature learning. However, ML’s interpretability better aligns with clinical demands for transparency, whereas DL’s reliance on large annotated datasets and computing resources limits its deployment in resource-constrained regions [64]. Third, validation strategies exposed generalizability limitations: external validation of sMRI+DL models showed reduced sensitivity (85% vs 90%) and specificity (91% vs 93%) compared to internal validation (*P*<.001).

The reduced diagnostic accuracy observed in externally validated models may stem from (1) data distribution shift—differences in feature distributions, class balance, and temporal trends between training and external datasets; (2) overfitting—models may have overfitted to training-specific noise or spurious patterns, limiting generalization; and (3) implementation and annotation inconsistencies—variations in data preprocessing, feature scaling, and labeling protocols across datasets [65].

### Future Directions and Study Limitations

This study has two methodological constraints: potential selection bias from English-only inclusion and possible overestimation by prioritizing optimal contingency tables. Future research should focus on enhancing evidence robustness through multinational, multiethnic cohorts; improving transparency via open-source preprocessing codes, findable, accessible, interoperable, and reusable-compliant data sharing, and independent validation; and balancing performance and interpretability via explainable DL frameworks to meet clinical ethical standards [66].

We also acknowledge the risk of publication bias, as studies with positive results are more likely to be published, particularly in AI-related research where rapid progress and selective reporting may skew the literature. Additionally, language and geographic biases may exist since only English-language articles were included and most studies originated from a limited number of regions (eg, China, the United States, and Europe). This may limit the generalizability of our findings to underrepresented regions.

Despite ^18^F-FDG PET’s higher accuracy, its radiation exposure and cost hinder widespread screening [67]. Conversely, sMRI’s cost-effectiveness and noninvasiveness position it as a first-line screening tool, with PET reserved for confirmatory testing in complex cases—a tiered diagnostic strategy. The 2023 Responsible AI for Social and Ethical Healthcare consensus implementation priorities, including transparent cost-benefit frameworks cross-modal standardization, and dynamic performance monitoring [68].

### Comparison With Existing Reviews

Although previous reviews have explored the diagnostic accuracy of AI in AD, studies involving meta-analyses remain scarce [69-71]. Conducting such meta-analyses faces significant challenges, particularly due to substantial methodological heterogeneity across studies. This heterogeneity manifests in multiple dimensions, including variations in neuroimaging modalities, disparities in model validation strategies, and differences in algorithm types—all of which influence AD diagnostic performance and complicate the synthesis of evidence.

Borchert et al [70] conducted a comprehensive systematic review of 255 neuroimaging studies utilizing AI for dementia diagnosis and prognosis. Their findings demonstrated that discriminative models, particularly DL approaches, outperformed algorithmic classifiers in distinguishing AD patients from healthy controls. However, they emphasized critical methodological limitations, with conclusions primarily relying on qualitative synthesis rather than quantitative evidence.

In a systematic review and meta-analysis by Sun et al [71], the diagnostic accuracy of DL models based on ^18^F-FDG PET for AD was investigated. While the study reported excellent diagnostic performance, notable heterogeneity was observed during meta-analysis, raising concerns about the reliability of the findings. Furthermore, the study focused exclusively on DL, overlooking the widespread application of traditional ML methods in current clinical research for AD diagnostic modeling.

In contrast, our meta-analysis incorporates a broader range of studies, rigorously controls methodological heterogeneity through stringent quality assessment and detailed subgroup analyses, and systematically evaluates the diagnostic accuracy of both ML and DL in AD. By emphasizing methodological rigor and the importance of external validation in AI-assisted neuroimaging for AD diagnosis, this study addresses critical gaps in the existing literature.

Although our subgroup comparisons between ML and DL models provide a useful overview of broad methodological trends, it must be noted that algorithm complexity, training data size, and model optimization procedures vary considerably within each group. The observed performance differences may, therefore, reflect not only model class but also differences in dataset size, feature representation, and implementation quality. Future studies should aim to compare individual algorithms under standardized conditions.

### Conclusions

In conclusion, AI can effectively support the diagnosis of AD using sMRI and ^18^F-FDG PET imaging. Among these approaches, combining PET imaging with DL techniques yields the highest diagnostic accuracy. These findings suggest that a future direction lies in integrating precision neuroimaging with AI tools. To bring such systems into routine clinical use—helping doctors detect AD earlier, personalize treatment, and improve patient outcomes—future studies should focus on repeated validation with high-quality clinical datasets and the development of standardized implementation protocols.

## Supplementary material

10.2196/76981 Multimedia Appendix 1 Detailed database search process, characteristics of included studies, quality assessment results, and subgroup analysis findings.

10.2196/76981 Checklist 1 PRISMA 2020 checklist.

## Abbreviations

^18^FDG-PET: fluorine-18 fluorodeoxyglucose positron emission tomography
AD: Alzheimer disease
AI: artificial intelligence
DL: deep learning
MeSH: Medical Subject Headings
ML: machine learning
PICOS: Population, Intervention, Comparison, Outcome, Study Design
PRISMA-DTA: Preferred Reporting Items for Systematic Reviews and Meta-Analyses of Diagnostic Test Accuracy
sMRI: structural magnetic resonance imaging
SROC-AUC: summary receiver operating characteristic curve area
TRIPOD-AI: Transparent Reporting of a Multivariable Prediction Model for Individual Prognosis or Diagnosis–Artificial Intelligence
