# Supplementary material for: Comparative Diagnostic Accuracy of AI-Assisted Fluorine-18 Fluorodeoxyglucose Positron Emission Tomography Versus Structural Magnetic Resonance Imaging in Alzheimer Disease: Systematic Review and Meta-Analysis
Source: JMIR Aging. 2025 Oct 8;8:e76981. doi: 10.2196/76981 (PMC12507341; doi:10.2196/76981)
Supplement: Multimedia Appendix 1 [file aging-v8-e76981-s001.docx]

**Table of Contents**

Table S1. Search terms and search strategy.

Table S2. Description of quality assessment based on TRIPOD-AI domains.

Table S3. Other characteristics of all included studies (n = 38).

Table S4. Tabular presentation of TRIPOD-AI.

Table S5. Summary estimates and meta-regression of the pooled performance for moderate-to-high-quality sMRI studies.

Table S1. Search terms and search strategy

| Database | Search strategy | Results |
| --- | --- | --- |
| PubMed (2006 to 2025 Jan 20) | #1 "alzheimer s diseases"[Title/Abstract] | 1295 |
|  | #2 "alzheimer syndrome"[Title/Abstract] | 23 |
|  | #3 "alzheimer type dementia"[Title/Abstract] | 776 |
|  | #4 "dementia alzheimer type"[Title/Abstract] | 50 |
|  | #5 "alzheimer diseases"[Title/Abstract] | 177 |
|  | #6 "alzheimers diseases"[Title/Abstract] | 1174 |
|  | #7 "alzheimer dementia"[Title/Abstract] | 840 |
|  | #8 "alzheimer dementias"[Title/Abstract] | 60 |
|  | #9 "dementia alzheimer"[Title/Abstract] | 1217 |
|  | #10 "alzheimer s disease"[Title/Abstract] | 179309 |
|  | #11 "dementia senile"[Title/Abstract] | 37 |
|  | #12 1 or 2 or 3 or 4 or 5 or 6 or 7or 8 or 9 or 10 or 11 | 181806 |
|  | #13 "f18 fluorodeoxyglucose"[Title/Abstract] | 192 |
|  | #14 "18f fluorodeoxyglucose"[Title/Abstract] | 5869 |
|  | #15 "fluorodeoxyglucose 18f"[Title/Abstract] | 756 |
|  | #16 "18F-FDG"[Title/Abstract] | 12323 |
|  | #17 "18FDG"[Title/Abstract] | 1953 |
|  | #18 "fdg"[Title/Abstract] | 48246 |
|  | #19 "fludeoxyglucose f 18"[Title/Abstract] | 135 |
|  | #20 "f 18 fludeoxyglucose"[Title/Abstract] | 20 |
|  | #21 "Fluorine-18-fluorodeoxyglucose"[Title/Abstract] | 2441 |
|  | #22 "fluorodeoxyglucose f 18"[Title/Abstract] | 170 |
|  | #23 "f 18 fluorodeoxyglucose"[Title/Abstract] | 1367 |
|  | #24 13 or 14 or 15 or 16 or 17 or 18 or 19 or 20 or 21 or 22 or 23 | 51368 |
|  | #25 "positron emission tomography"[Title/Abstract] | 81735 |
|  | #26 "pet imaging"[Title/Abstract] | 17339 |
|  | #27 "imaging pet"[Title/Abstract] | 735 |
|  | #28 "pet scan"[Title/Abstract] | 5379 |
|  | #29 "pet scans"[Title/Abstract] | 5991 |
|  | #30 "scan pet"[Title/Abstract] | 126 |
|  | #31 "pet"[Title/Abstract] | 146723 |
|  | #32 25 or 26 or 27 or 28 or 29 or 30 or 31 | 169387 |
|  | #33 24 and 32 | 49123 |
|  | #34 "magnetic resonance imaging"[Title/Abstract] | 334010 |
|  | #35 "nmr imaging"[Title/Abstract] | 1280 |
|  | #36 "imaging nmr"[Title/Abstract] | 59 |
|  | #37 "mri scans"[Title/Abstract] | 15798 |
|  | #38 "magnetic resonance images"[Title/Abstract] | 12773 |
|  | #39 "MRI"[Title/Abstract] | 366049 |
|  | #40 34 or 35 or 36 or 37 or 38 or 39 | 546678 |
|  | #41 "functional magnetic resonance imaging"[Title/Abstract] | 42875 |
|  | #42 "fMRI"[Title/Abstract] | 63421 |
|  | #43 "magnetic resonance imaging functional"[Title/Abstract] | 105 |
|  | #44 "functional mri"[Title/Abstract] | 17016 |
|  | #45 41 or 42 or 43 or 44 | 85139 |
|  | #46 40 not 45 | 487138 |
|  | #47 "artificial intelligence"[Title/Abstract] | 72354 |
|  | #48 "computer reasoning"[Title/Abstract] | 8 |
|  | #49 "machine intelligence"[Title/Abstract] | 392 |
|  | #50 "computational intelligence"[Title/Abstract] | 599 |
|  | #51 "machine learning"[Title/Abstract] | 145271 |
|  | #52 "deep learning"[Title/Abstract] | 83342 |
|  | #53 47 or 48 or 49 or 50 or 51 or 52 | 252226 |
|  | #54 "Diagnosis"[Title/Abstract] | 2102262 |
|  | #55 "Diagnose"[Title/Abstract] | 109000 |
|  | #56 "Examination"[Title/Abstract] | 857477 |
|  | #57 "detection"[Title/Abstract] | 1229100 |
|  | #58 "accuracy"[Title/Abstract] | 622846 |
|  | #59 "sensitivity"[Title/Abstract] | 1100848 |
|  | #60 "specificity"[Title/Abstract] | 614542 |
|  | #61 "area under the receiver operating characteristic curve"[Title/Abstract] | 25110 |
|  | #62 "AUC"[Title/Abstract] | 155905 |
|  | #63 "SROC-AUC"[Title/Abstract] | 59 |
|  | #64 54 or 55 or 56 or 57 or 58 or 59 or 60 or 61 or 62 or 63 | 5253952 |
|  | #65 12 and 33 and 53 and 64 | 89 |
|  | #66 12 and 46 and 53 and 64 | 787 |
| Wed of Science (Search in: All Databases; Collections: All) (Timespan=All years) | #1 TS=("alzheimer s diseases" OR "alzheimer syndrome" OR "alzheimer type dementia" OR "dementia alzheimer type" OR "alzheimer diseases" OR "alzheimers diseases" OR "alzheimer dementia" OR "alzheimer dementias" OR "dementia alzheimer" OR "alzheimer s disease" OR "dementia senile") and Preprint Citation Index (Exclude – Database) | 263941 |
|  | #2 TS=("fdg" OR "f18 fluorodeoxyglucose" OR "18f fluorodeoxyglucose" OR "fluorodeoxyglucose 18f" OR "18F-FDG" OR "18FDG" OR "fludeoxyglucose f 18" OR "f 18 fludeoxyglucose" OR "Fluorine-18-fluorodeoxyglucose" OR "fluorodeoxyglucose f 18" OR "f 18 fluorodeoxyglucose") and Preprint Citation Index (Exclude – Database) | 89828 |
|  | #3 TS=("positron emission tomography" OR "pet imaging" OR "imaging pet" OR "pet scan" OR "pet scans" OR "scan pet" OR "pet") and Preprint Citation Index (Exclude – Database) | 368736 |
|  | #4 TS=("artificial intelligence" OR "computer reasoning" OR "machine intelligence" OR "computational intelligence" OR "machine learning" OR "deep learning") and Preprint Citation Index (Exclude – Database) | 1367635 |
|  | #5 TS=("Diagnosis" OR "Diagnose" OR "Examination" OR "detection" OR "accuracy" OR "sensitivity" OR "specificity" OR "area under the receiver operating characteristic curve" OR "AUC" OR "SROC-AUC") and Preprint Citation Index (Exclude – Database) | 14052059 |
|  | #6 TS=("magnetic resonance imaging" OR "nmr imaging" OR "imaging nmr" OR "mri scans" OR "magnetic resonance images" OR "MRI") and Preprint Citation Index (Exclude – Database) | 1056147 |
|  | #7 TS=("functional magnetic resonance imaging" OR "fMRI" OR "magnetic resonance imaging functional" OR "functional mri") and Preprint Citation Index (Exclude – Database) | 136732 |
|  | #8 (#6) NOT #7 and Preprint Citation Index (Exclude – Database) | 942246 |
|  | #9 #1 AND #2 AND #3 AND #4 AND #5 and Preprint Citation Index (Exclude – Database) | 230 |
|  | #10 #2 AND #4 AND #5 AND #8 and Preprint Citation Index (Exclude – Database) | 2380 |
| Embase (1974 to 2024 Jun 20) | #1 ("alzheimer s diseases" OR "alzheimer syndrome" OR "alzheimer type dementia" OR "dementia alzheimer type" OR "alzheimer diseases" OR "alzheimers diseases" OR "alzheimer dementia" OR "alzheimer dementias" OR "dementia alzheimer" OR "alzheimer s disease" OR "dementia senile"):ab,ti AND (("fdg" OR "f18 fluorodeoxyglucose" OR "18f fluorodeoxyglucose" OR "fluorodeoxyglucose 18f" OR "18F-FDG" OR "18FDG" OR "fludeoxyglucose f 18" OR "f 18 fludeoxyglucose" OR "Fluorine-18-fluorodeoxyglucose" OR "fluorodeoxyglucose f 18" OR "f 18 fluorodeoxyglucose"):ab,ti) AND (("positron emission tomography" OR "pet imaging" OR "imaging pet" OR "pet scan" OR "pet scans" OR "scan pet" OR "pet"):ab,ti) AND (("artificial intelligence" OR "computer reasoning" OR "machine intelligence" OR "computational intelligence" OR "machine learning" OR "deep learning"):ab,ti) AND (("Diagnosis" OR "Diagnose" OR "Examination" OR "detection" OR "accuracy" OR "sensitivity" OR "specificity" OR "area under the receiver operating characteristic curve" OR "AUC" OR "SROC-AUC"):ab,ti) | 111 |
|  | #2 ("magnetic resonance imaging" OR "nmr imaging" OR "imaging nmr" OR "mri scans" OR "magnetic resonance images" OR "MRI"):ab,ti NOT (("functional magnetic resonance imaging" OR "fMRI" OR "magnetic resonance imaging functional" OR "functional mri"):ab,ti) AND (("alzheimer s diseases" OR "alzheimer syndrome" OR "alzheimer type dementia" OR "dementia alzheimer type" OR "alzheimer diseases" OR "alzheimers diseases" OR "alzheimer dementia" OR "alzheimer dementias" OR "dementia alzheimer" OR "alzheimer s disease" OR "dementia senile"):ab,ti) AND (("artificial intelligence" OR "computer reasoning" OR "machine intelligence" OR "computational intelligence" OR "machine learning" OR "deep learning"):ab,ti) AND (("Diagnosis" OR "Diagnose" OR "Examination" OR "detection" OR "accuracy" OR "sensitivity" OR "specificity" OR "area under the receiver operating characteristic curve" OR "AUC" OR "SROC-AUC"):ab,ti) | 821 |

Table S2. Description of quality assessment based on TRIPOD-AI domains

|  | **Q1** | **Q2** | **Q3** | **Q4** | **Q5** | **Q6** | **Q7** | **Q8** | **Q9** |
| --- | --- | --- | --- | --- | --- | --- | --- | --- | --- |
|  | **Data quality** | **Data preprocessing** | **Model construction** | **Validation methods** | **Performance metrics** | **Model interpretability** | **Clinical relevance** | **Bias control** | **Novelty and limitations** |
| **Score: 0** | No description of data sources or sample size < 100 | No description of preprocessing steps | No clarification of algorithm type or parameter settings | Only use the training set for testing | Only report accuracy | No provision of model interpretability | Only technical metrics evaluated | Presence of data leakage or selection bias | Lack of novelty and no discussion of limitations |
| **Score: 1** | Sample size of 100–200 with description of basic demographic characteristics | Description of basic preprocessing (such as standardization/denoising) | Reasonable selection of DL/ML algorithms with description of the architecture | Adopt cross-validation (≥5 folds) or an independent validation set | Report AUC/sensitivity/specificity/F1 score | Use tools such as Grad-CAM/SHAP to visualize key regions | Conduct correlation analysis with clinical scales | Specify exclusion criteria and control for confounding factors | Proposing improved methods |
| **Score: 2** | Sample size > 200, including multicenter data and complete clinical/imaging data | Detailed explanation of registration/segmentation/feature extraction methods, and provision of quality control metrics | Integration of prior medical knowledge for model optimization | Multicenter external validation plus time-series validation | Report additional positive predictive value (PPV)/negative predictive value (NPV)/Dice coefficient | Incorporate pathological mechanisms to explain feature importance | Provide diagnostic decision curves | Implement blinded assessment or adversarial validation | Comparing advantages with existing methods and proposing a clinical translation roadmap |

Table 3. Other characteristics of all included studies (n = 38).

| **First author and year** | **Participant characteristics** | | | | **Data characteristics** | | | **AI Characteristics** | | | | | **Performance Metrics** |
| --- | --- | --- | --- | --- | --- | --- | --- | --- | --- | --- | --- | --- | --- |
|  | **Definition** | **Mean age (SD) years** | **Percentage of female participants** | **MMSE (Mean ± SD))** | **Modality** | **Internal validation?** | **External validation?** | **AI Model** | **DL/ML** | **GM/DM** | **Transfer learning?** | **Data augmentation?** | **SEN/SEP/ACC/AUC** |
| Zhang (2011) | 51-AD;52-NC | AD:75.2(7.4) NC:75.3(5.2) | AD:35% NC:35% | AD:23.8(2.0) NC:29.0(1.2) | sMRI | Yes (10-fold CV) | No | SVM | ML | DM | NR | NR | 0.86/0.86/0.86/- |
|  |  |  |  |  | PET |  |  |  |  |  |  |  | 0.86/0.87/0.87/- |
| Yun (2015) | 71-AD;85-NC | AD:75.2(7.0) NC:75.8(4.5) | AD:42% NC:36% | AD:23.3(2.2) NC:28.9(1.1) | sMRI | Yes (LOOCV) | No | LDA | ML | DM | NR | NR | 0.78/0.91/0.85/0.91 |
|  |  |  |  |  | PET |  |  |  |  |  |  |  | 0.83/0.92/0.88/0.94 |
| Westman (2012) | 96-AD;111-NC | AD:74.4(7.8) NC:75.6(5.2) | AD:43%  NC:50% | AD:23.5(1.8) NC:29.1(0.9) | sMRI | Yes (7-fold CV) | No | OPLS | ML | DM | NR | NR | 0.83/0.90/0.87/0.93 |
| Vemuri (2008) | 140-AD;140-NC | AD:78(NR) NC:77(NR) | AD:43% NC:43% | AD:22(NR) NC:29(NR) | sMRI | Yes (4-fold CV) | No | SVM | ML | DM | NR | NR | 0.86/0.86/-/- |
|  | 50-AD;50-NC | AD:78.5(NR) NC:79(NR) | AD:32% NC:34% | AD:20(NR) NC:29(NR) |  | Yes (Hold-out) |  |  |  |  |  |  | 0.86/0.86/-/- |
| Suk (2014) | 93-AD;101-NC | AD:75.5(7.4) NC:75.9(4.8) | AD:39% NC:39% | AD:23.5(2.1) NC:28.9(1.1) | sMRI | Yes (10-fold CV) | No | DBM; SVM | ML+DL | GM+DM | NR | NR | 0.92/0.95/0.92/0.97 |
|  |  |  |  |  | PET |  |  |  |  |  |  |  | 0.88/0.96/0.92/0.98 |
| Sayeed (2002) | 18-AD;10-NC | NR | NR | NR | PET | Yes (LOOCV) | No | DFA | ML | DM | NR | NR | 0.94/0.90/0.93/- |
| Pan (2019) | 247-AD;246-NC | AD:75.1(8.0) NC:74.4(6.1) | AD:42% NC:48% | AD:23.2(2.1) NC:29.0(1.2) | PET | Yes (10-fold CV) | No | SVM | ML | DM | NR | NR | 0.92/0.95/0.93/0.97 |
| Padilla (2012) | 53-AD;52-NC | NR | NR | NR | PET | Yes (LOOCV) | No | SVM | ML | DM | NR | NR | 0.88/0.85/0.87/- |
| Ni (2021) | 118-AD;149-NC | AD:76.2(7.5) NC:76.3(5.8) | AD:40% NC:42% | AD:21.7(5.4) NC:28.5(4.0) | PET | Yes (Hold-out) | No | CNN | DL | DM | Yes | Yes | 0.93/0.91/0.92/0.97 |
| Magnin (2009) | 16-AD;22-NC | AD:74.1(5.2) NC:72.3(5.0) | AD:69% NC:82% | AD:23.1(2.9) NC:28.5(1.3) | sMRI | Yes (bootstrap resampling) | No | SVM | ML | DM | NR | NR | 0.92/0.97/0.95/- |
| Lu (2018) | 226-AD;304-NC | AD:75.1(7.9) NC:73.5(6.0) | AD:41% NC:51% | AD:23.2(2.1) NC:29.1(1.2) | PET | Yes (10-fold CV) | No | MDNN | DL | DM | NR | NR | 0.92/0.95/0.94/- |
| Liu (2012) | 198-AD;229-NC | AD:75.7(7.7) NC:76.0(5.0) | AD:47% NC:48% | AD:23.3(2.0) NC:29.1(1.0) | sMRI | Yes (10-fold CV) | No | SRC | ML | DM | NR | NR | 0.86/0.95/0.91/0.95 |
| Liu (2018) | 93-AD;100-NC | AD:75.5(7.4) NC:76.0(4.8) | AD:39% NC:39% | AD:23.5(2.1) NC:28.9(1.1) | PET | Yes (10-fold CV) | No | 2D-CNN | DL | DM | NR | NR | 0.91/0.91/0.91/0.95 |
| Li (2015) | 25-AD;30-NC | AD:72(6) NC:74(5) | AD:40% NC:30% | AD:23.2(2.2) NC:28.6(1.4) | PET | Yes (10-fold CV) | No | GMM; SVM | ML | GM+DM | NR | NR | 0.92/0.86/0.89/0.97 |
|  | 30-AD;16-NC | AD:69(8) NC:66(6) | AD:40% NC:56% | AD:21.5(5.2) NC:29.3(0.7) |  |  |  |  |  |  |  |  | 0.94/0.90/0.92/0.97 |
| Lerch (2008) | 19-AD;17-NC | AD:68.8(6.9) NC:61.0(9.1) | NR | AD:21.2(NR) NC:29.3(NR) | sMRI | Yes (LOOCV) | No | QDA | ML | DM | NR | NR | 0.94/0.95/0.94/- |
| Kim (2020) | 141-AD;348-NC | AD:75.9(7.9) NC:76.3(6.4) | AD:35% NC:50% | NR | PET | Yes (NR) | No | CNN | DL | DM | Yes | NR | 0.88/0.94/0.91/- |
|  | 80-AD;72-NC | AD:71.1(9.3) NC:63.3(9.3) | AD:38% NC:54% |  |  | N/A | Yes |  |  |  |  |  | 0.80/0.93/0.86/- |
| Kim (2020) | 139-AD;347-NC | AD:76.0(7.9) NC:76.3(6.4) | AD:65% NC:50% | AD:27.1(1.8) NC:29.0(1.4) | PET | Yes (NR) | No | BEGA; SVM | ML+DL | GM+DM | NR | NR | 0.92/0.97/0.95/0.98 |
|  | 73-AD;68-NC | AD:70.8(9.5) NC:63.5(9.1) | AD:60% NC:56% | AD:21.7(4.5) NC:29.3(1.0) |  | N/A | Yes |  |  |  |  |  | 0.92/0.97/0.94/0.98 |
| Katako (2018) | 94-AD;111-NC | AD:75.5(8.3) NC:75.3(6.4) | AD:37% NC:NR | AD:24.2(1.8) NC:29.0(1.1) | PET | Yes (10-fold CV) | No | SVM | ML | DM | NR | NR | 0.84/0.96/-/0.95 |
| Ismail (2023) | 511-AD;535-NC | NR | NR | NR | sMRI | Yes (10-fold CV) | No | MultiAz-Net; SVM | ML+DL | DM | Yes | Yes | 0.93/0.91/0.91/- |
|  |  |  |  |  | PET |  |  |  |  |  |  |  | 0.94/0.93/0.94/- |
| Illan (2011) | 95-AD;97-NC | AD:77.3(7.4) NC:76.7(5.2) | NR | NR | PET | Yes (LOOCV) | No | SVM | ML | DM | NR | NR | 0.88/0.89/0.88/- |
| Hinrichs (2009) | 89-AD;94-NC | AD:76.1(7.0) NC:75.8(4.5) | AD:40% NC:37% | AD:21.7(3.0) NC:29.0(0.8) | sMRI | Yes (Leave-many-out CV) | No | LPBoosting | ML | DM | NR | NR | 0.85/0.80/0.82/0.88 |
|  |  |  |  |  | PET |  |  |  |  |  |  |  | 0.84/0.82/0.84/0.87 |
| Gray (2012) | 50-AD;54-NC | NR | AD:40% NC:33% | AD:23.5(2.0) NC:28.9(1.2) | PET | Yes (Monte Carlo CV) | No | SVM | ML | DM | NR | NR | 0.83/0.94/0.88/- |
| Gray (2013) | 37-AD;35-NC | AD:76.8(6.6) NC:74.5(5.2) | AD:38% NC:34% | AD:23.5(2.0) NC:28.9(1.2) | sMRI | Yes (Monte Carlo CV) | No | RF | ML | DM | NR | NR | 0.89/0.76/0.83/- |
|  |  |  |  |  | PET |  |  |  |  |  |  |  | 0.86/0.87/0.86/- |
| Feng (2019) | 93-AD;100-NC | NR | NR | NR | sMRI | Yes (10-fold CV) | No | FSBi-LSTM;3D-CNN | DL | DM | NR | NR | 0.93/0.92/0.93/0.95 |
|  |  |  |  |  | PET |  |  |  |  |  |  |  | 0.93/0.91/0.92/0.97 |
| Cuingnet (2011) | 68-AD;81-NC | AD:76.2(7.2) NC:76.5(5.2) | AD:51% NC:53% | AD:23.2(2.1) NC:29.2(0.9) | sMRI | Yes (Hold-out) | No | SVM | ML | DM | NR | NR | 0.81/0.95/-/- |
| Chen (2022) | 183-AD,229-NC | AD:75.6(7.6) NC:76.2(5.1) | AD:48% NC:48% | AD:23.1(2.5) NC:29.2(1.0) | sMRI | N/A | Yes | 2D-CNN;3D-CNN | DL | DM | NR | NR | 0.86/0.88/0.86/0.91 |
|  | 143-AD,184-NC | AD:75.6(7.8) NC:77.3(6.7) | AD:41% NC:53% | AD:21.9(3.8) NC:28.8(1.7) |  |  |  |  |  |  |  |  | 0.89/0.91/0.91/0.95 |
| Song (2021) | 95-AD,126-NC | AD:76.5(7.0) NC:75.3(5.8) | AD:43% NC:44% | AD:18.6(4.2) NC:29.6(0.7) | sMRI | Yes (10-fold CV) | No | 3D-CNN | DL | DM | NR | NR | 0.86/0.92/0.90/- |
|  |  |  |  |  | PET |  |  |  |  |  |  |  | 0.89/0.94/0.92/- |
| Li (2019) | 130-AD,162-NC | AD:71.3(6.1) NC:72.7(6.1) | AD:46% NC:50% | NR | PET | Yes (Monte Carlo CV) | No | SVM | ML | DM | NR | NR | 0.93/0.90/0.91/0.91 |
|  | 22-AD,22-NC | AD:57.3（6.5） NC:57.3（6.5） | AD：27% NC：27% |  |  | N/A | Yes |  |  |  |  |  | 0.91/0.91/0.92/0.93 |
| Ahila (2022) | 220-AD;635-NC | AD:75(NR) NC:70(NR) | AD:40% NC:30% | AD:NR NC:NR | PET | Yes (Hold-out) | No | 2D-CNN | DL | DM | NR | NR | 0.94/0.96/0.97 |
| Toussaint（2012） | 80-AD;80-NC | AD:76.0(6.3) NC:76.4(4.6) | AD:44% NC:36% | AD:23.7(2.1) NC:29.0(1.1) | PET | Yes (LOOCV) | No | SVM | ML | DM | NR | NR | 0.90/0.95/0.92/- |
| Tong (2014) | 198-AD;231-NC | AD:75.7(7.7) NC:76.0(5.0) | AD:48% NC:48% | AD:23.3(2.0) NC:29.1(1.0) | sMRI | Yes (LOOCV) | No | SVM | ML | DM | NR | NR | 0.85/0.93/0.89 |
| Min (2014) | 97-AD;128-NC | AD:75.9(6.8) NC:76.1(5.1) | AD:51% NC:51% | AD:23.4(1.8) NC:29.1(1.0) | sMRI | Yes (10-fold CV) | No | SVM | ML | DM | NR | NR | 0.89/0.94/0.92/0.87 |
| Jin (2020) | 261-AD;231-NC | NR | NR | NR | sMRI | Yes (Leave center out CV) | No | 3D Attention Network | DL | DM | NR | NR | 0.87/0.96/0.91/0.94 |
|  | 261-AD;231-NC |  |  |  |  | N/A | Yes |  |  |  |  |  | 0.79/0.96/0.87/0.91 |
|  | 227-AD;305-NC | AD:74.8(7.6) NC:74.6(5.7) |  | AD:22.0(3.5) NC:29.1(1.2) |  | Yes (10-fold CV) | No |  |  |  |  |  | 0.89/0.94/0.92/0.94 |
|  | 227-AD;305-NC |  |  |  |  | N/A | Yes |  |  |  |  |  | 0.88/0.85/0.86/0.91 |
| Cho (2012) | 66-AD;80-NC | AD:76.0(7.1) NC:76.2(5.4) | AD:47% NC:46% | AD:23.3(2.0) NC:29.2(1.0) | sMRI | Yes (Hold-out) | No | LDA | ML | DM | NR | NR | 0.82/0.93/-/- |
| Chincarini (2011) | 144-AD;189-NC | AD:75.5(7.5) NC:76.6(5.1) | AD:46% NC:50% | AD:22.3(3.3) NC:29.1(0.9) | sMRI | Yes (20-fold CV) | No | RF; SVM | ML | DM | NR | NR | 0.89/0.94/-/0.97 |
| Beheshti (2017) | 92-AD;94-NC | AD:75.3(6.5) NC:73.4(5.7) | AD:54% NC:53% | AD:23.5(2.5) NC:29.7(3.9) | sMRI | Yes (10-fold CV) | No | SVM | ML | DM | NR | NR | 0.89/0.97/0.93/0.94 |
| Anandh (2016) | 30-AD;55-NC | AD:78.1(7.0) NC:67.1(5.5) | AD:67% NC:69% | AD:21.2(4.1) NC:29.2(1.1) | sMRI | Yes (10-fold CV) | No | SVM | ML | DM | NR | NR | 0.99/0.97/0.98/- |
| Amoroso (2018) | 38-AD;29-NC | AD:73.6(8.0) NC:75.0(6.3) | AD:47% NC:45% | AD:22.7(2.3) NC:29.0(0.9) | sMRI | Yes (5-fold CV) | No | RF | ML | DM | NR | NR | 0.90/0.88/0.88/- |
|  | 48-AD;52-NC | AD:78.4(6.0) NC:74.8(6.0) | AD:46% NC:48% | AD:24.3(1.9) NC:29.0(0.8) |  | Yes (Hold-out) |  |  |  |  |  |  | 0.96/0.74/0.86/- |

^a^AD: alzheimer's disease.

^b^NC: normal cognitive.

^c^sMRI: structural magnetic resonance imaging.

^d^SVM: support vector machine.

^e^RF: random forest.

^f^LDA: linear discriminant analysis.

^g^CNN: convolutional neural network.

^h^LP: linear program.

^i^FSBi-LSTM: fully stacked bidirectional long short-term memory.

^j^BEGAN; boundary equilibrium generative adversarial network.

^k^OPLS; orthogonal partial least squares.

^l^DBM: deep boltzmann machine.

^m^QDA: quadratic discriminant analysis.

^n^GMM: gaussian mixture model.

^o^SRC: sparse representation-based classifier.

^p^MDNN: multiscale deep neural network.

^q^DFA: discriminant function analysis.

^r^CV: cross validation.

^s^LOOCV: leave one out cross validation.

^t^ML: machine learning.

^u^DL: deep learning.

^v^GM: generative model.

^w^DM: discriminative model.

^x^NR: no report.

Table S4. Tabular presentation of TRIPOD-AI

| **First author and year** | **Q1** | **Q2** | **Q3** | **Q4** | **Q5** | **Q6** | **Q7** | **Q8** | **Q9** | **Total score** | **High (16–18) Medium (10–15) Low (0–9)** |
| --- | --- | --- | --- | --- | --- | --- | --- | --- | --- | --- | --- |
| Zhang (2011) | 2 | 2 | 2 | 1 | 2 | 1 | 1 | 1 | 2 | 14 | Medium |
| Yun (2015) | 2 | 2 | 2 | 1 | 1 | 1 | 1 | 1 | 2 | 13 | Medium |
| Westman (2012) | 2 | 2 | 2 | 2 | 2 | 1 | 2 | 2 | 2 | 17 | High |
| Vemuri (2008) | 2 | 1 | 1 | 2 | 1 | 0 | 1 | 1 | 1 | 10 | Medium |
| Suk (2014) | 2 | 2 | 2 | 1 | 2 | 2 | 1 | 1 | 2 | 15 | Medium |
| Sayeed (2002) | 0 | 1 | 1 | 1 | 0 | 0 | 0 | 0 | 1 | 4 | Low |
| Pan (2019) | 2 | 2 | 2 | 1 | 1 | 1 | 1 | 1 | 1 | 12 | Medium |
| Padilla (2012) | 1 | 1 | 1 | 1 | 1 | 0 | 0 | 0 | 0 | 5 | Low |
| Ni (2021) | 2 | 2 | 2 | 1 | 1 | 0 | 1 | 1 | 1 | 11 | Medium |
| Magnin (2009) | 0 | 1 | 1 | 1 | 0 | 0 | 0 | 1 | 1 | 5 | Low |
| Lu（2018） | 2 | 2 | 2 | 1 | 1 | 1 | 1 | 1 | 1 | 12 | Medium |
| Liu (2012) | 2 | 2 | 2 | 2 | 2 | 1 | 2 | 2 | 2 | 17 | High |
| Liu (2018) | 2 | 1 | 2 | 2 | 2 | 2 | 2 | 1 | 2 | 16 | High |
| Li (2015) | 2 | 1 | 1 | 1 | 1 | 0 | 1 | 1 | 1 | 9 | Low |
| Lerch (2008) | 0 | 2 | 1 | 1 | 1 | 1 | 1 | 0 | 1 | 8 | Low |
| Kim (2020) | 2 | 2 | 2 | 2 | 1 | 2 | 2 | 2 | 2 | 17 | High |
| Kim (2020) | 2 | 2 | 2 | 2 | 2 | 1 | 2 | 2 | 2 | 17 | High |
| Katako (2018) | 2 | 1 | 1 | 1 | 1 | 0 | 1 | 1 | 1 | 9 | Low |
| Ismail (2023) | 2 | 2 | 2 | 1 | 2 | 2 | 2 | 2 | 2 | 17 | High |
| Illan (2011) | 2 | 2 | 1 | 1 | 1 | 1 | 1 | 1 | 1 | 11 | Medium |
| Hinrichs (2009) | 1 | 1 | 2 | 1 | 1 | 1 | 1 | 1 | 2 | 11 | Medium |
| Gray (2012) | 2 | 1 | 1 | 1 | 1 | 1 | 1 | 1 | 1 | 10 | Medium |
| Gray (2013) | 2 | 2 | 1 | 2 | 2 | 1 | 2 | 1 | 2 | 15 | Medium |
| Feng (2019) | 2 | 1 | 2 | 1 | 1 | 1 | 1 | 1 | 2 | 12 | Medium |
| Cuingnet (2011) | 2 | 1 | 1 | 1 | 1 | 1 | 1 | 1 | 1 | 10 | Medium |
| Chen (2022) | 2 | 2 | 2 | 2 | 1 | 1 | 1 | 1 | 2 | 14 | Medium |
| Song (2021) | 2 | 2 | 1 | 1 | 1 | 1 | 1 | 1 | 2 | 12 | Medium |
| Li (2019) | 2 | 2 | 2 | 1 | 2 | 2 | 2 | 2 | 2 | 17 | High |
| Ahila (2022) | 2 | 1 | 1 | 1 | 1 | 0 | 0 | 0 | 1 | 7 | Low |
| Toussaint (2012) | 2 | 2 | 1 | 1 | 2 | 1 | 2 | 2 | 2 | 15 | Medium |
| Tong (2014) | 2 | 1 | 1 | 1 | 1 | 0 | 1 | 1 | 2 | 10 | Medium |
| Min (2014) | 1 | 1 | 1 | 1 | 1 | 0 | 0 | 1 | 1 | 7 | Low |
| Jin (2020) | 2 | 2 | 2 | 2 | 2 | 2 | 2 | 1 | 2 | 17 | High |
| Cho (2012) | 2 | 1 | 2 | 1 | 1 | 1 | 1 | 0 | 2 | 11 | Medium |
| Chincarini (2011) | 2 | 1 | 1 | 1 | 2 | 1 | 2 | 1 | 1 | 12 | Medium |
| Beheshti (2017) | 2 | 1 | 1 | 1 | 1 | 0 | 0 | 1 | 2 | 9 | Low |
| Anandh (2016) | 1 | 1 | 1 | 0 | 1 | 0 | 0 | 0 | 1 | 5 | Low |
| Amoroso (2018) | 2 | 2 | 2 | 2 | 2 | 1 | 2 | 2 | 2 | 17 | High |

Table S5. Summary estimates and meta-regression of the pooled performance for moderate-to-high-quality sMRI studies.

| **Main directory** | **Subordinate directory** | **No. of tables** | **Sensitivity (%)** | | **Specificity (%)** | | **Joint p value** | **AUC (95%CI)** |
| --- | --- | --- | --- | --- | --- | --- | --- | --- |
|  |  |  |  |  |  |  |  |  |
|  |  |  | SE (95% CI) | I^2^ | SP (95% CI) | I^2^ |  |  |
| **sMRI+ML** | Traditional ensemble learning | 4 | 89 (83, 94) | 14.76 | 79 (73, 84) | 6.17 | ＜0.01 | 0.89 (0.86, 0.91) |
|  | Non-traditional ensemble learning | 10 | 87 (85, 90) | 17.47 | 93 (90, 95) | 57.65 |  | 0.93 (0.90, 0.95) |
| **sMRI+DL** | Internal validation | 6 | 90 (88, 92) | 54.36 | 93 (91, 95) | 38.60 | ＜0.01 | 0.97 (0.95, 0.98) |
|  | External validation | 4 | 85 (81, 89) | 72.26 | 91 (85, 94) | 83.65 |  | 0.93 (0.90, 95) |

^a^sMRI: structural magnetic resonance imaging.

^b^ML: machine learning.

^c^DL: deep learning.
